# High‐dose therapy followed by autologous stem cell transplantation emerges as the preferred salvage therapy in patients with limited‐stage Hodgkin lymphoma progressing/relapsing after initial therapy: A subset analysis of the EORTC/LYSA/FIL H10 trial

**DOI:** 10.1002/hem3.70105

**Published:** 2025-04-02

**Authors:** Gotti Manuel, Yana Stepanishyna, Tetiana Skrypets, Luigi Marcheselli, Caterina Cristinelli, Barbara Botto, Sanne Tonino, Doriane Cavalieri, Alessandro Pulsoni, Martin Hutchings, Mohammad Hammoud, Catherine Fortpied, Luigi Rigacci, Wouter Plattel, Marc André, Massimo Federico

**Affiliations:** ^1^ Division of Hematology Fondazione IRCCS Policlinico San Matteo Pavia Italy; ^2^ Department of Hematology Jules Bordet Institute Brussels Belgium; ^3^ Hematology and Cell Therapy Unit IRCCS Istituto Tumori “Giovanni Paolo II” Bari Italy; ^4^ FIL Trial Office Modena Italy; ^5^ Hemato‐Oncology Department Saint‐Louis Hospital Paris France; ^6^ Division of Hematology Ematologia AOU Città della Salute e della Scienza Torino Italy; ^7^ Department of Hematology University Medical Centers Amsterdam, University of Amsterdam Amsterdam The Netherlands; ^8^ Department of Hematology Lille University Hospital Lille France; ^9^ Dipartimento di Medicina Traslazionale e di Precisione Università La Sapienza, Roma and UOC Ematologia e Trapianto Ospedale S. Maria Goretti Latina Italy; ^10^ Department of Hematology Rigshospitalet, University of Copenhagen Copenhagen Denmark; ^11^ Hematology Department Hospital Henri‐Mondor AP‐HP Creteil France; ^12^ Statistics Department EORTC Headquarters Brussels Belgium; ^13^ FIL Hematology, Campus Bio‐Medico University Rome Italy; ^14^ EORTC University Medical Center Groningen Groningen The Netherlands; ^15^ Department of Hematology CHU UCL Namur Yvoir Belgium; ^16^ CHIMOMO Department University of Modena and Reggio Emilia Modena Italy

Long‐term survival of patients with limited‐stage classical Hodgkin lymphoma (cHL) is excellent, with more than 90% surviving and disease‐free for 10 years after diagnosis and initial treatment.^1^, ^2^ Nevertheless, improving patient outcomes and minimizing the risk of long‐term toxicities continue to be priorities.

Early response assessment with positron emission tomography (ePET) is an important predictor of outcomes, and several trials have focused on response‐adapted treatments to avoid the need for radiotherapy (RT) in patients with an early complete metabolic response. In the EORTC/LYSA/FIL H10 intergroup randomized trial, such a response‐adapted strategy resulted in the achievement of 95% overall survival at 10 years.^1^ However, in the 1419 cases with updated follow‐up, 106 progressions or recurrences (7.5%) were recorded.

In this study, we report the results of a detailed analysis of second‐line treatment choices and outcomes in these 106 patients. Previously untreated patients aged 15–70 years with classic supradiaphragmatic stage I or II cHL were eligible for the EORTC/LYSA/FIL H10 trial. Both favorable (F) and unfavorable (U) patients according to the EORTC criteria were included. All patients received two cycles of doxorubicin, bleomycin, vinblastine, and dacarbazine (ABVD), after which an ePET was performed. The primary objective of this study was to evaluate whether involved‐node RT could be omitted without loss of efficacy in ePET‐negative patients.^2^ This long‐term analysis was restricted to the subset of patients with progression/recurrence after study entry. The principal endpoints of this analysis were survival after recurrence (SAR) and progression‐free survival after recurrence (PFS2). From November 2006 to June 2011, 1950 patients were enrolled in the EORTC/LYSA/FIL H10 trial by 158 institutions, in which 1925 completed two ABVD cycles and performed an ePET scan. Long‐term follow‐up has been updated for 1419 of these patients (Supporting Information S3: Data Supplement Figure 1).

Overall, 106 (7.5%) events were recorded, including 17 progressions (events within 6 months from study entry, 1.2%) and 89 recurrences (6.3%). Ninety‐five events occurred in this study population before the safety amendment, and 11 thereafter. Patients' characteristics and outcomes for the entire cohort are summarized in Table 1.

**Table 1:** Patient characteristics (*N* = 106).

Characteristics	
Age, years	
Median (IQR)	32 (20–61)
Range	18–69
>45 years, *n* (%)	22 (21)
Sex, *n* (%)	
Female	49 (46)
ePET‐2 status, *n* (%)	
ePET‐2‐positive	36 (34)
Stage at registration, *n* (%)	
I	16 (25)
II	90 (85)
First‐line treatment, *n* (%)	
4 ABVD	26 (24.5)
3 ABVD + INRT	2 (1.9)
6 ABVD	34 (32)
4 ABVD + INRT	32 (30.2)
2 AVBD + 2 escBEACOPP + INRT	12 (11.3)
IPS at registration, *n* = 81 (%)	
3–7	36 (44)
Risk group, *n* (%)	
Unfavorable	72 (68)
Type of event, *n* (%)	
Refractory (<6 months)	17 (16)
Relapse (>6 months)	89 (84)

Abbreviations: ABVD, doxorubicin, bleomycin, vinblastine, and dacarbazine; escBEACOPP, bleomycin, etoposide, doxorubicin, cyclophosphamide, vincristine, procarbazine, and prednisone; ePET‐2, early positron emission tomography 2 cycles of ABVD; INRT, involved‐node radiotherapy; IPS, International Prognostic Score.

Events occurred in 5.5% (28/508), 6.5% (42/651), and 13.8% (36/260) of patients with early favorable ePET‐, early unfavorable ePET‐, and ePET‐positive disease, respectively.

Sites of progression/recurrence were recorded in 32 (30.2%) patients with initially involved and non‐irradiated sites, 12 (11.3%) involved and irradiated, 45 (42.4%) not initially involved and not irradiated, and 17 (16.0%) not involved but irradiated sites. The 17 progressions occurred after a median of 4.4 months from study entry (range 2.1–6.0 months). With respect to the 89 recurrences, 28 (31.5%) occurred in the standard treatment arm and 61 (68.5%) in the experimental (chemotherapy alone) arm.

After a median follow‐up of 9.5 years, the cumulative incidence of failure (progression/recurrence) was 7.6% (Supporting Information S3: Data Supplement Figure 2). Salvage therapy consisted of ABVD in 5 patients, escBEACOPP (bleomycin, etoposide, doxorubicin, cyclophosphamide, vincristine, procarbazine, and prednisone) in 11, intensified chemotherapy in 85, and unknown in the remaining 5. In the group of patients treated with intensified chemotherapy, 65 patients received dexamethasone, cytarabine, and cisplatin (DHAP), or ifosfamide, carboplatin, and etoposide (ICE), or ifosfamide, mitoxantrone, and etoposide (MINE); 16 received ifosfamide, gemcitabine, vinorelbine, and prednisone (IGEV) or IGEV‐like, and the remaining 6 received either ifosfamide, etoposide, and cytarabine (IVA) or ifosfamide, etoposide, and oxaliplatin (IVOX). Forty‐one patients were further consolidated with high‐dose therapy followed by ASCT, while the remaining 44 were not (Figure 1). No significant differences were observed between the two groups (Supporting Information S1: Data Supplement Table 1).

**Figure 1:**
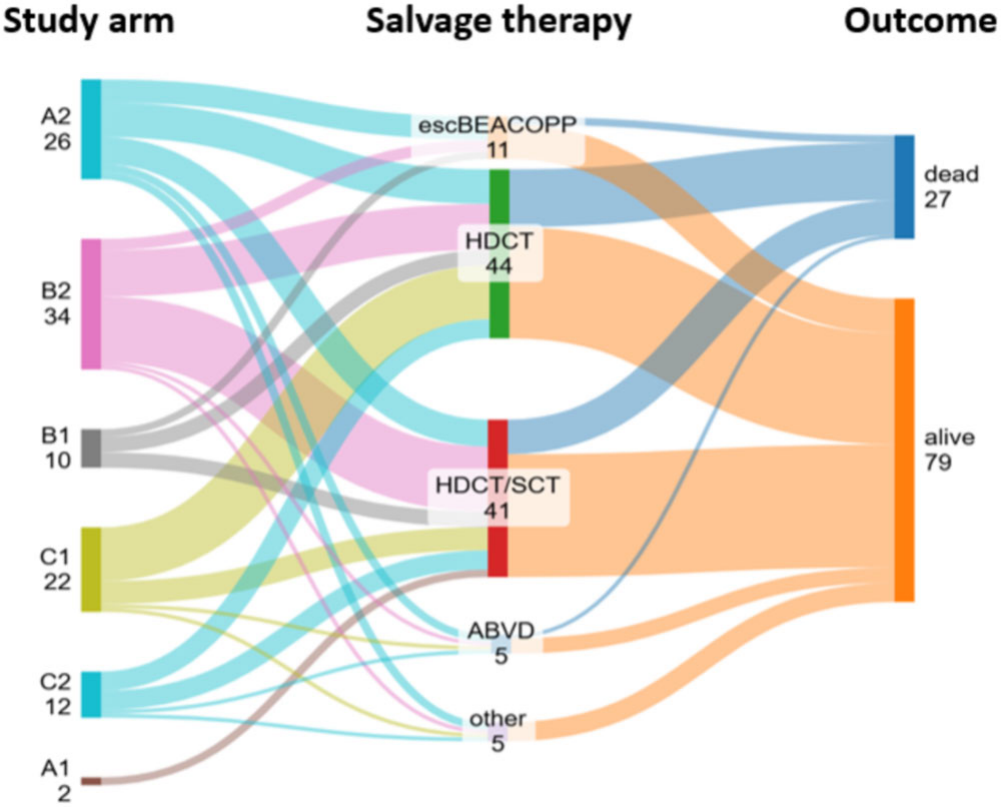
**Sankey diagram**. ABVD, doxorubicin, bleomycin, vinblastine, and dacarbazine; BEACOPP, bleomycin, etoposide, doxorubicin, cyclophosphamide, vincristine, procarbazine, and prednisone; HDCT, high‐dose chemotherapy; INRT, involved‐node radiotherapy; SCT, stem cell transplant; A1, 3 ABVD + INRT; A2, 3 ABVD; B1, 4‐ABVD ± INRT; B2, 6 ABVD; C1, 3 or 4 ABVD + INRT; C2, 2 AVBD + 2 escBEACOPP + INRT.

Intensified chemotherapy was administered to 13/16 (81.2%) patients with refractory and 72/85 (84.7%) with relapsed disease (*p* = 0.715). Of note, no patient was rescued with RT. At the last follow‐up, 79 patients were alive; 27 had died due to disease progression (16; 59%), second malignancy (1; 3.7%), cardiovascular disease (1; 3.7%), or toxicity related to second or further salvage therapy (3; 11.1%). In six cases (22.2%), the cause of death was unknown.

Ten‐year SAR and PFS2 were 71% (95% CI: 59%–80%) and 37% (95% CI: 27%–48%), respectively (Supporting Information S3: Data Supplement Figure 3). Ten‐year SAR was 75% and 94% in the favorable group of patients who received combined modality treatment (F&CMT) or chemotherapy alone (F&C), respectively (*p* = 0.254); 65% and 81% in the unfavorable group with combined modality treatment (U&CMT) or chemotherapy alone (U&C), respectively (*p* = 0.438); and 67% and 55% (at 9.5 years) in the ePET treated with ABVD or escBEACOPP, respectively (*p* = 0.311) (Supporting Information S3: Data Supplement Figure 4).

Ten‐year SAR was 80%, 89%, 65%, and 74% for patients treated with ABVD, escBEACOPP, intensified chemotherapy, and intensified chemotherapy followed by ASCT, respectively. In the group of patients treated with intensified regimens, the 5‐year SAR was 85% and 68% for those receiving ASCT or not (*p* = 0.121). PFS2 was 49% with ASCT and 31% without (*p* = 0.190), showing no significant difference (Supporting Information S3: Data Supplement Figure 5).

In univariate analysis, only patients classified as refractory due to time to progression within 6 months from randomization exhibited a significantly poorer outcome, irrespective of the type of second‐line therapy (Supporting Information S2: Data Supplement Table 2). Ten‐year SAR was 50% and 75% for patients with refractory and relapsed disease, respectively (log‐rank *p* = 0.009) (Supporting Information S3: Data Supplement Figure 6).

The H10 trial was designed to achieve a satisfactory balance between the risks and benefits of early PET response‐adapted strategy in limited‐stage classical HL. Using this approach, 1.2% of patients progressed within 6 months from the start of initial therapy, and 6.3% relapsed. Overall, these results compare more than favorably with the published data.^1^, ^2^, ^3^, ^4^, ^5^, ^6^, ^7^, ^8^

In this study, we report what we believe is the largest case series of patients that had relapse/refractory disease and necessitated second‐line therapy after homogeneous initial treatment. Surprisingly, no patient was treated with RT as second‐line therapy. Events occurred in 5.1%, 6.8%, and 13.1% of patients with favorable ePET‐negative, unfavorable ePET‐negative, and ePET‐positive HL, respectively.

Intensified chemotherapy was by far the most preferred treatment choice, with more than 80% of patients treated with DHAP/DHAP‐like, ICE, MINE, IGEV/IGEV‐like, or IVA; half consolidated with HDT and ASCT, which proves to be an effective treatment for relapse of HL, especially after initial limited‐stage disease.^9^, ^10^, ^11^ Two randomized phase III studies, conducted by the British National Lymphoma Investigation^12^ and the GHSG/European Group for Blood and Marrow Transplantation,^13^ compared HDT/ASCT with conventional chemotherapy in patients with relapsed or refractory HL. Both studies showed significant improvements in EFS, PFS, and FFTF (with no difference in OS) for patients with relapsed or refractory HL who underwent HDT/ASCR compared to conventional chemotherapy alone.

The PET‐oriented approach tested in H10 is in line with that reported in the RAPID and HD16 trials.^3^, ^7^ However, based on our results, whether sparing first‐line RT in most patients with limited‐stage HL is cost‐effective or not remains unknown. It is still debated whether it is better to offer a very toxic and debilitating second‐line treatment to the few patients who relapse or to offer consolidation RT to all patients, sparing most of them from intensive second‐line therapy.

When the H10 trial was designed and conducted, no new drugs were available, and intensified chemotherapy followed by ASCT was one of the best chances of cure to be offered to patients needing second‐line treatment. Currently, whether relapsing patients still require transplant as part of second‐line therapy, and whether the introduction of brentuximab vedotin (BV),^14^ nivolumab,^15^ or pembrolizumab,^16^, ^17^ used alone or in combination with CT,^18^, ^19^, ^20^ reduces the need for transplant remain unanswered questions. The available data suggest that new drugs have improved the response to second‐line therapy, but whether high‐dose therapy and ASCT can subsequently be spared is still unclear.

In summary, second‐line systemic therapy followed by HDT/ASCT is still the preferred approach for patients with relapsed or refractory classical HL. However, the observed cumulative incidence of relapse and the long‐term PFS and OS after salvage therapy highlight the need for continued efforts to optimize frontline approaches and develop more effective salvage strategies using novel agents for patients with high‐risk diseases.

## AUTHOR CONTRIBUTIONS

Gotti Manuel, Catherine Fortpied, and Marc André designed the research project. Gotti Manuel, Luigi Marcheselli, and Catherine Fortpied conducted the statistical analysis. All authors reviewed the results and contributed to data interpretation. Gotti Manuel, Yana Stepanishyna, Massimo Federico, Tetiana Skrypets, Catherine Fortpied, Luigi Marcheselli, Wouter Plattel, and Marc André wrote the manuscript. All authors critically reviewed, commented, and approved the final manuscript.

## CONFLICT OF INTEREST STATEMENT

The authors declare no conflicts of interest.

### FUNDING

This study was funded by the Associazione Angela Serra, Mont Godinne Foundation, Lymphoma Study Association (LYSA), Fondazione Italiana Linfomi, and European Organisation for Research and Treatment of Cancer. Y. S. is the recipient of the EHA Ukraine Bridge Funding Program.

## Supporting information

Supporting information.

Supporting information.

Supporting information.

## Data Availability

The data that support the findings of this study are openly available in the EORTC data release policy at https://www.eortc.org/data-sharing/.
