# Supplementary material for: High‐dose therapy followed by autologous stem cell transplantation emerges as the preferred salvage therapy in patients with limited‐stage Hodgkin lymphoma progressing/relapsing after initial therapy: A subset analysis of the EORTC/LYSA/FIL H10 trial
Source: Hemasphere. 2025 Apr 2;9(4):e70105. doi: 10.1002/hem3.70105 (PMC11962756; doi:10.1002/hem3.70105)
Supplement: Supplementary file 1 — Supporting information. [file HEM3-9-e70105-s001.docx]

**Data Supplement Table 1. Patient characteristics in groups receiving salvage therapy and salvage therapy +ASCT.**

| **Factor** | **HDT** | **HDT+BMT** | **p-value** |
| --- | --- | --- | --- |
|  | **n (%)** | |  |
| Age at R/R, mean (SD) | 34 (1.8) | 36 (2.0) | 0.425 |
| Age at R/R >45y | 8 (18) | 11 (27) | 0.437 |
| Sex M | 23 (52) | 22 (54) | 1.000 |
| LDH >ULN | 34 (79) | 29 (74) | 0.794 |
| Bulky disease, yes | 23 (66) | 16 (44) | 0.096 |
| Unfavorable risk | 33 (75) | 30 (73) | 1.000 |
| Stage II | 38 (86) | 34 (83) | 0.767 |
| iPET positive | 20 (45) | 11 (27) | 0.114 |
| Refractory | 8 (18) | 5 (12) | 0.552 |

Fisher’s exact probability; * t-test.

LDH, Bulky, risk group and stage: characteristics at diagnosis. R/R: refractory/relapse; LDH: lactate dehydrogenase; ULN: upper limit of normality.
