# Supplementary material for: High‐dose therapy followed by autologous stem cell transplantation emerges as the preferred salvage therapy in patients with limited‐stage Hodgkin lymphoma progressing/relapsing after initial therapy: A subset analysis of the EORTC/LYSA/FIL H10 trial
Source: Hemasphere. 2025 Apr 2;9(4):e70105. doi: 10.1002/hem3.70105 (PMC11962756; doi:10.1002/hem3.70105)
Supplement: Supplementary file 2 — Supporting information. [file HEM3-9-e70105-s002.docx]

**Data Supplement Table 2. Five-year SAR and prognostic variables: univariate analysis.**

| **Factor** |  | **5-yr SAR (95%CI)** | **Log rank, p** |
| --- | --- | --- | --- |
| Age | <45 | 78 (67-86) |  |
|  | ≥45 | 80 (55-92) | 0.883 |
| Sex | M | 74 (60-84) |  |
|  | F | 83 (69-91) | 0.177 |
| Histology | NS | 77 (66-84) |  |
|  | Other | 84 (62-94) | 0.504 |
| Time to progression | <24 mo | 75 (65-83) |  |
|  | 24+ mo | 89 (62-97) | 0.408 |
| **Time to progression** | **<6 mo** | **59 (33-78)** |  |
|  | **6+ mo** | **82 (72-89)** | **0.009** |
| Risk | Favorable | 97 (80-99) |  |
|  | Unfavorable | 69 (57-79) | 0.081 |
| IPS | 0-2 | 79 (64-89) |  |
|  | 3-7 | 78 (59-89) | 0.471 |
| iPET2 | - | 85 (73-91) |  |
|  | + | 66 (48-79) | 0.288 |

Abbreviations: M, male; F, female; NS, nodular sclerosis; ePET-2, early positron emission tomography; IPS, International Prognostic Score; SAR, survival after relapse; CI, confidence interval.
