# Supplementary material for: High‐dose therapy followed by autologous stem cell transplantation emerges as the preferred salvage therapy in patients with limited‐stage Hodgkin lymphoma progressing/relapsing after initial therapy: A subset analysis of the EORTC/LYSA/FIL H10 trial
Source: Hemasphere. 2025 Apr 2;9(4):e70105. doi: 10.1002/hem3.70105 (PMC11962756; doi:10.1002/hem3.70105)
Supplement: Supplementary file 3 — Supporting information. [file HEM3-9-e70105-s003.docx]

**Data Supplement Figure 1.**

**
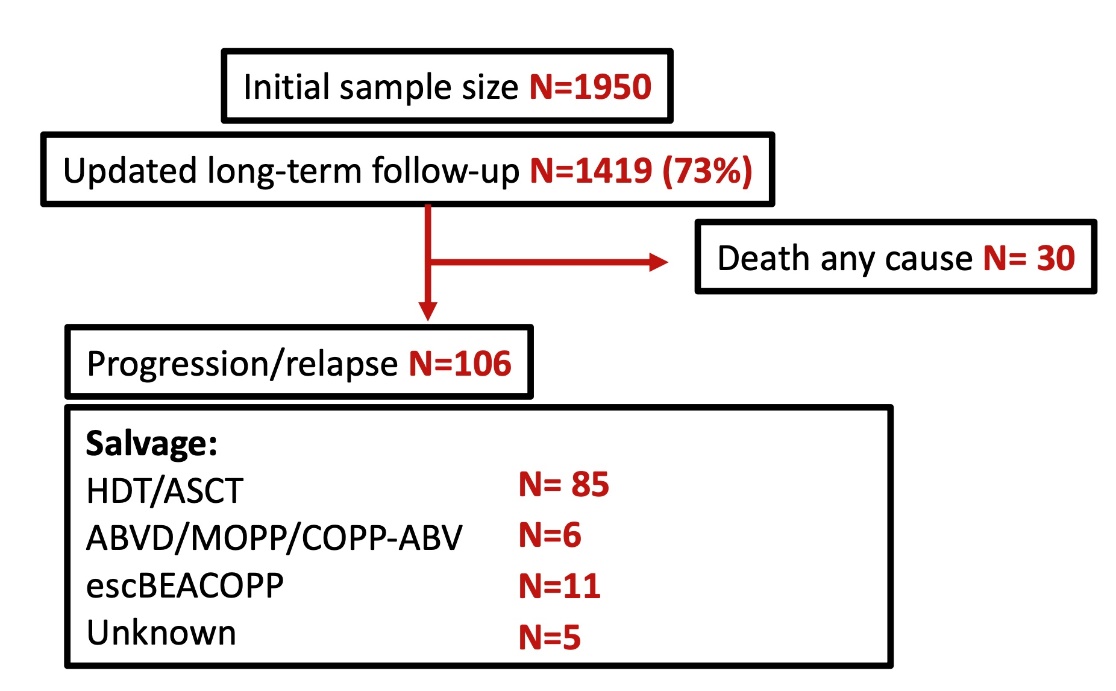


Data Supplement Figure 1**. CONSORT flow diagram. HDT, high-dose therapy; ASCT, autologous stem cell transplant; ABVD, doxorubicin, bleomycin, vinblastine, and dacarbazine; MOPP, nitrogen mustard, vincristine, procarbazine, and prednisone; COPP, cyclophosphamide, vincristine, prednisone, procarbazine; escBEACOPP, bleomycin, etoposide, doxorubicin, cyclophosphamide, vincristine, procarbazine, and prednisone.

**Data Supplement Figure 2.**

**Data Supplement Figure 2**. Cumulative incidence of failure (progression/recurrence) after median follow-up 9.5 years

**Data Supplement Figure 3.**


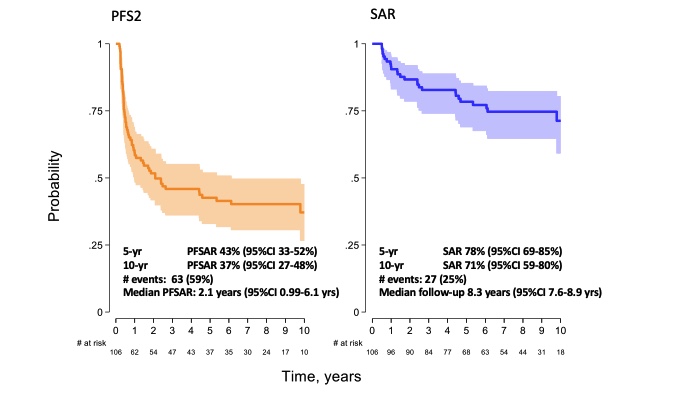


**Data Supplement Figure 3.** Ten-year SAR and PFS after second-line therapy. SAR, survival after relapse; PFS2, progression-free survival after relapse.

**Data Supplement Figure 4.**

**Data Supplement Figure 4**. Ten-year survival after relapse (SAR) for patients initially treated in the different study arms. Fav, favorable patients; Unf, unfavorable; CMT, combined modality treatment; CT, chemotherapy alone; iPET, interim positron emission tomography; ABVD, doxorubicin, bleomycin, vinblastine, and dacarbazine; BEACOPP, bleomycin, etoposide, doxorubicin, cyclophosphamide, vincristine, procarbazine, and prednisone.

**Data Supplement Figure 5.**

**
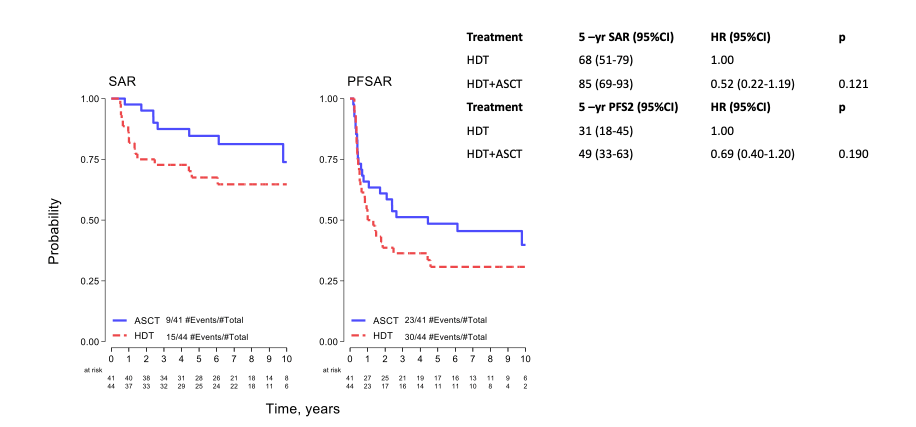

Data Supplement Figure 5**. Ten-year SAR and PFS2 by ASCT vs. HDT Alone (n=85). SAR, survival after relapse; PFS2, progression-free survival after relapse; ASCT, autologous stem cell transplant; HDT, high-dose therapy; HR, hazard ratio.

**Data Supplement Figure 6.**

**Data Supplement Figure 6.** Ten-year SAR for patients with progression before and after 6 months. SAR, survival after relapse; HR, hazard ratio. Relapse 10-yr SAR 75% (95%CI: 61–85%); Refractory 10-yr SAR 50% (95%CI: 24–72%); HR 2.90 (95%CI: 1.26–6.67), p = 0.012 (log-rank, p = 0.009).
